# Adaptive limitations of white spruce populations to drought imply vulnerability to climate change in its western range

**DOI:** 10.1111/eva.12845

**Published:** 2019-08-05

**Authors:** Zihaohan Sang, Jaime Sebastian‐Azcona, Andreas Hamann, Annette Menzel, Uwe Hacke

**Affiliations:** ^1^ Department of Renewable Resources University of Alberta Edmonton AB Canada; ^2^ Department of Ecology and Ecosystem Management Technical University of Munich Freising Germany; ^3^ Institute for Advanced Study Technical University of Munich Garching Germany

**Keywords:** boreal forest, climate change, dendroecology, drought resilience, ecological genetics, *Picea glauca*, provenance trials

## Abstract

A cost‐effective climate change adaptation strategy for the forestry sector is to move seed sources to more northern and higher elevation planting sites as part of ongoing reforestation programs. This is meant to match locally adapted populations with anticipated environments, but adaptive traits do not always show population differences suitable to mitigate climate change impacts. For white spruce, drought tolerance is a critical adaptive trait to prevent mortality and productivity losses. Here, we use a 40‐year‐old provenance experiment that has been exposed to severe drought periods in 1999 and 2002 to retrospectively investigate drought response and the adaptive capacity of white spruce populations across their boreal range. Relying on dendrochronological analysis under experimentally controlled environments, we evaluate population differences in resistance, resilience, and recovery to these extreme events. Results showed evidence for population differentiation in resistance and recovery parameters, but provenances conformed to approximately the same growth rates under drought conditions and had similar resilience metrics. The lack of populations with better growth rates under drought conditions is contrary to expectations for a wide‐ranging species with distinct regional climates. Populations from the wettest environments in the northeastern boreal were surprisingly drought‐tolerant, suggesting that these populations would readily resist water deficits projected for the 2080s, and supporting the view that northeastern Canada will provide a refugium for boreal species under climate change. The findings also suggest that white spruce is sensitive to growth reductions under climate change in the western boreal. The study highlights that population differentiation in adaptive capacity is species‐ and trait‐specific, and we provide a counterexample for drought tolerance traits, where assisted migration prescriptions may be ineffective to mitigate climate change impacts. For resource managers and policy makers, we provide maps where planning for widespread declines of boreal white spruce forests may be unavoidable.

## 1 INTRODUCTION

Since the beginning of the 20th century, mean annual temperature across the North American boreal forest has increased by 1.5 to 2.5°C, significantly exceeding the mean global temperature increase (Romero‐Lankao et al., 2014). In the western boreal, climate conditions have also become drier by around 10% in mean annual precipitation (Mbogga, Hamann, & Wang, 2009; Romero‐Lankao et al., 2014). Warmer temperatures and reduced precipitation together have induced widespread impacts on the boreal forests and its ecosystems due to drought stress (Price et al., 2013). Across large regions, where water deficits limit growth, reduced forest productivity has been documented for western boreal tree species (Chen et al., 2017; Chhin & Wang, 2008; Hogg, Barr, & Black, 2013) or in some cases has caused significant dieback and mortality (Allen et al., 2010; Michaelian, Hogg, Hall, & Arsenault, 2011; Peng et al., 2011; Worrall et al., 2013).

White spruce (*Picea glauca* (Moench) Voss) is one of the most common and widely distributed boreal forest species in North America. The species is also commercially important and comprises roughly a quarter of the Canadian Forest Inventory (NRC, 2013). Dendrochronological and inventory plot‐based research has identified the species as sensitive to growth reductions under warming and increased moisture deficits expected under climate change projections. Water deficits limit the range of white spruce at the southern fringe (Chhin & Wang, 2008; Chhin, Wang, & Tardif, 2004), droughts caused significant mortality of white spruce (Peng et al., 2011), Barber, Juday, and Finney (2000), Hogg, Michaelian, Hook, and Undershultz (2017) showed that moisture stress reduced growth due to recent climate trends, and D'Orangeville et al. (2018) predict that white spruce is more sensitive to growth reductions under increased temperature than other boreal species.

To address negative impacts of climate change and associated extreme events on natural and managed forest ecosystems, several general options are available (Millar, Stephenson, & Stephens, 2007). Adaptive strategies for forest management include the protection of highly valued resources, resilience options to improve the capacity of ecosystems to recover after disturbance, and management response to facilitate the transition of ecosystems from current to new conditions. A cost‐effective climate change adaptation strategy for the forestry sector is to move seed sources to more northern and higher elevation planting sites as part of ongoing reforestation programs (Aitken & Bemmels, 2016; Lenoir, Gegout, Marquet, de Ruffray, & Brisse, 2008; Pedlar et al., 2012). This is meant to match locally adapted populations with anticipated environments, but adaptive traits do not always show population differences suitable to mitigate climate change impacts.

A commonly used approach to study local adaptation of populations, the adaptive capacity of a species, and genetic variability in adaptive traits is provenance and progeny field trials. In these experiments, seed sources from different origins, sometimes with a known pedigree, are grown in a common garden to reveal intra‐species genetic variance. Provenance and progeny trials for white spruce have been evaluated for disease resistance (Alfaro, He, Kiss, King, & Yanchuk, 1996), wood quality (Beaulieu, Girard, & Fortin, 2002), and survival and growth (Gray et al., 2016; Li, Beaulieu, & Bousquet, 1997; Lu et al., 2014). Resistance to drought and other climate extreme events are not normally reported from provenance experiments, but they have been assessed in natural stands through tree ring analysis (Fritts, 1974; Fritts, Vaganov, Sviderskaya, & Shashkin, 1991; Jacoby & D'Arrigo, 1995). Using tree ring analysis in common garden experiments is a useful, new research approach that combines the strength of historical biology analysis with population genetic and genomic inferences (George et al., 2017; Housset et al., 2018; Montwé, Isaac‐Renton, Hamann, & Spiecker, 2016; Montwé, Spiecker, & Hamann, 2015).

Here, we contribute a dendrochronological analysis of white spruce provenances from throughout the range sources of the species, grown for almost four decades in a common garden experiment at a centrally located test site that already experienced above‐average water deficits compared to the species range under normal conditions. The test site has been exposed to severe drought periods in 1999 and 2002 that caused significant growth reductions and mortality of tree species in the region, offering a unique opportunity to study drought response of genotypes in a common garden environment where environmental variation is uniform and experimentally controlled. Specifically, (1) we quantify drought resistance, recovery, and resilience of boreal spruce populations from throughout the range; (2) we evaluate drought tolerance traits in the context of long‐term growth performance and survival; and (3) we interpret correlations between drought tolerance and growth metrics in the context of the origin climate of provenances to infer local adaptation. The results are discussed in the context of sensitivity to growth reductions under climate change and possible climate change adaptation strategies to maintain health and productivity of one of the ecologically and commercially most important boreal forest tree species.

## 2 METHODS

### 2.1 Plant material and sampling design

White spruce seedlings from 43 provenances across Canada were planted at a common garden trial near Calling Lake, Alberta, (55°17’N, 113°09’W, 625 m ASL). Planting stock consisted of containerized seedlings, germinated in 1978 and planted in 1982. This field trial is a randomized complete block design with 5 blocks, 5‐tree row plots with 2.5 m × 2.5 m spacing. Two border rows were planted to minimize edge effects. Tree height and survival were evaluated at age 32, and the diameter at breast height (DBH), approx. 1.3 m above the ground, was measured at age 27.

**Figure 1:**
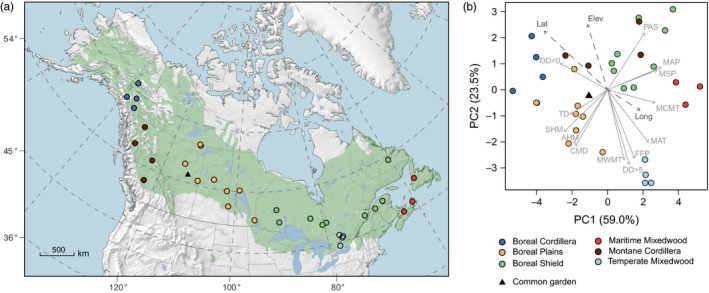
Location of white spruce provenances and test sites (a) and their climatic conditions (b). The 33 provenances are assigned to geographic regions with similar climate that approximately correspond to the Canadian ecozone classification system. The vectors in the principal component analysis show how provenances and regions are associated with climate variables. Climate variables include mean annual precipitation (MAP), mean summer precipitation (MSP), precipitation as snow (PAS), mean annual temperature (MAT), mean warmest month temperature (MWMT), mean coldest month temperature (MCMT), growing degree‐days above 5°C (DD > 5), continentality (TD), chilling degree‐days (DD < 0), frost‐free period (FFP), annual and summer heat‐moisture index (AHM, SHM), as well as Hargreaves climatic moisture deficit (CMD). Latitude, longitude, and elevation are represented as dashed arrows and were not part of the ordination. The species range of white spruce is shown in green

For dendrochronological analysis, we used a subsampling design with 33 of 43 provenances. Where the original sampling design had local clusters of samples, sometimes from near‐identical locations, we selected one provenance to arrive at a well‐distributed geographical and climatic representation of growing conditions throughout the range of the species (Figure 1). Within each block of the experimental design, one tree was randomly selected per provenance (5 blocks × 33 provenances = 165 trees in total). The relatively small sample size was chosen to minimize potential injuries on these valuable genetic trials and was based on a power analysis of the number of samples needed to detect population differences in growth and drought tolerance traits. Two cores were sampled in 2017 from each individual at north and south sides of the stem with an increment borer at approximately 0.5 m stem height to capture growth data since the late 1980s while coring well above areas of growth influenced by the root collar. Ring width values from the two cores per tree were averaged prior to statistical analysis.

### 2.2 Dendrochronology analysis

Core samples were preserved in plastic straws with slices for air circulation. After air‐drying for two weeks, a total of 324 increment cores were glued in wooden cores mounts (six cores were too broken to reconstruct). Then, cores were prepared using a belt sander with progressively finer grains (400, 240, 100 grains per inch) and scanned at 6,400 DPI (Epson Perfection V800 Pro). Scans were analyzed with WinDENDRO software, version 2016a (Regent Instruments Canada Inc.). Tree ring series were cross‐dated in WinDENDRO, and errors such as missing rings were corrected prior to generating final cross‐dating statistics with the program COFECHA (Grissino‐Mayer, 2001; Holmes, 1983).

To evaluate the cross‐dating reliability of the complete chronology (from 1988 to 2017), we calculated the expressed population signal (EPS) according to Wigley, Briffa, and Jones (1984) for all 165 individuals. An exceptionally high value (EPS = 0.99) suggested near‐perfect cross‐dating, but this is an expected result for trees grown in a randomized common garden where environmental variation is experimentally controlled. In natural stands, an EPS of 0.85 is considered sufficiently consistent (Speer, 2010).

Cross‐dated chronologies were then subjected to detrending, because ring width is controlled not only by growing conditions but also by the age of the tree, with the ring widths near the pith being largest. In standard dendrochronological research, this age‐related trend is removed by subtracting a spline function fitted to each chronology. The residual after detrending is then converted to a standardized chronology with a mean of one and interannual growth variation expressed in units of standard deviations for further analysis (Cook, Briffa, Shiyatov, & Mazepa, 1990). However, fitting individual splines to trees grown in a common garden experiment for detrending might partially remove population differences. We therefore used a common spline function that has the same shape for all trees. Further, in our common garden trial, where all trees have the same age and were grown under controlled environments, the absolute growth value carries important information. We therefore did not work with standardized ring widths, but reversed the standardization after detrending using the original average and standard deviation of each tree ring chronology. The detrending was implemented with a smoothing parameter of 0.7, a relatively stiff spline, fitted to the combined normalized chronology data with the *smooth.spline()* function of the stats package (R Core Team, 2018).

To quantify white spruce resilience to the two drought events, we calculated indices for resistance, recovery, and resilience (Lloret, Keeling, & Sala, 2011). Resistance was calculated as the ratio of the radial growth during the drought over growth before the drought (higher resistance value indicates a small growth reduction during the event). Recovery was measured as the mean postdisturbance growth divided by the growth during the drought year (high recovery values indicate a strong rebound of growth after disturbance). The resilience index is the ratio of growth performance after and before the disturbance to quantify permanent damage (a resilience value below one suggests the tree failed to recover to the predisturbance performance after the drought).

In our common garden experiment, the two‐year period between the first and second drought events was not long enough for trees to fully recover from the very first drought event. We therefore used five years before the first drought (1993–1997) and five years after the second drought (2003–2007) as predisturbance and postdisturbance reference periods for calculation of indices for both drought events.

### 2.3 Climatic characterization

Climate data of seed source origins were obtained using the software ClimateNA v5.21 (Wang, Hamann, Spittlehouse, & Carroll, 2016). We used the climate period 1961–1990 as a representation of normal climate conditions. The period is a compromise between weather station coverage and representing climate conditions to which populations are putatively adapted. Prior to 1961, weather station coverage becomes sparse leading to inaccurate climate estimates, and subsequent to 1990, there is a significant anthropogenic warming signal. Provenances were grouped into six regions considering geographic location of the provenances (Figure 1a) as well as climate conditions summarized by a principal component analysis (Figure 1b). The groups approximately corresponded to the Canadian ecoregion classifications (Boreal Cordillera, Boreal Plains, Boreal Shield, Maritime Mixedwood, Montane Cordillera, and Temperate Mixedwood), and we use these names throughout the text when referring to populations of white spruce within these regions. Principal component analysis was implemented with the *princomp* () function of the stats package using the correlation matrix. We also generate maps of current and projected water deficits for the range of white spruce with the same ClimateNA v5.21 software package, using gridded data publicly available at http://tinyurl.com/ClimateNA.

To more precisely characterize growing conditions at the planting site, we obtained daily weather records from a meteorological station in Calling Lake (Station ID 3061117), at approximately 4 km distance to the planting site, operated by the Government of Canada (http://climate.weather.gc.ca). The weather data included daily average, maximum, and minimum temperature (°C) and the daily amount of snow and precipitation (mm). We calculated water deficits on a daily scale, based on a reference evapotranspiration (ET_0_) estimated with Hargreaves and Samani (1985) method, also used by the ClimateNA v5.21 software package.

### 2.4 Statistical analysis

Taking advantage of the randomized complete block design, regional means of drought response metrics and field measurements were estimated with a mixed‐model approach and best linear unbiased estimates (BLUEs) for the average and standard error of each region. For measurements of height and DBH, we evaluated 25 trees per provenance planted in 5‐tree row plots with a linear mixed model with blocks and plots within blocks specified as random effects. Regions were specified as a fixed factor, and provenances nested within regions were also treated as a random effect. The analysis was implemented with the *asreml*() and *predict*() functions of the asreml package (Gilmour, Gogel, Cullis, Welham, & Thompson, 2015) based on the following linear model:

$$Y_{\text{ijklm}}=\mu +R_{\text{i}}+\text{Prov}\;{\left(R\right)}_{\text{ij}}+B_{\text{k}}+\text{Plot}\;{\left(B\right)}_{\text{lk}}+e_{\text{ijlkm}}$$

where *Y* is the measurement of individual *m* of provenance *j* from region *i* planted in plot *l* within block *k*. The overall mean is denoted as μ and the experimental error as *e*. The model effects *R*, Prov*(R)*, *B*, and Plot*(B)* denote region, provenance within region, block, and plot within block, respectively.

The survival rate of white spruce from each region was estimated by fitting generalized linear mixed models (GLMM) for binomial data with the *glmer*() function of the lme4 package (Bates, Machler, Bolker, & Walker, 2015). Since only one tree per provenance of each block was sampled for drought resilience analysis, our linear mixed models for drought indices specify regions as fixed effects and blocks as random effects. For multiple pairwise statistical comparisons among regions, we corrected the α‐value with a Tukey adjustment, implemented with the *cld*() function of emmeans package (Lenth, 2018). The residuals of each mixed model were visually checked for meeting assumptions of the respective distributions of errors.

Associations among climate of origin and provenance means of traits measured in the common garden experiment were assessed with the nonparametric Spearman correlation coefficient, implemented with the *cor.test()* function of the R base package (R Core Team, 2018). Significance of correlations was adjusted for multiple inference using Holm's method (Rice,^1989^), implemented with the *adjust()* function of the R base package.

## 3 RESULTS

### 3.1 Chronologies and test site climate

**Figure 2:**
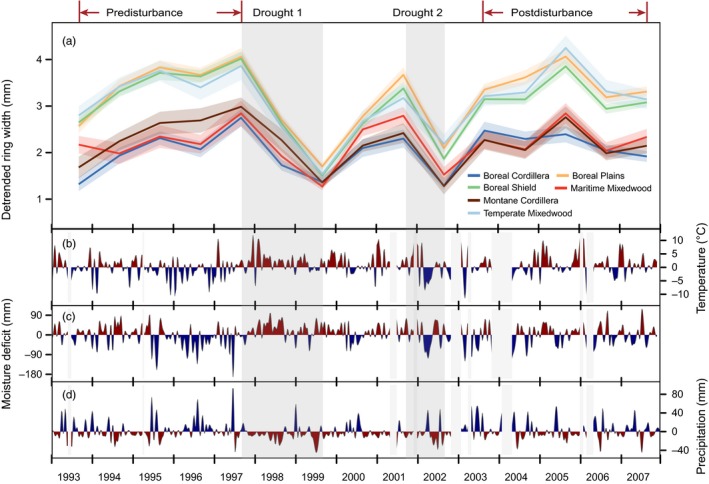
Detrended ring width increments (a) of provenances summarized by regions as shown in Figure 1. The standard error of each group is represented by colored transparent bands. Panels b, c, and d show anomalies in temperature, moisture deficit, and precipitation relative to the normal climate conditions of the test site. The integral from the zero value shown as colored area visualizes the severity of extreme climate events, integrating the size and length of the anomaly. Two drought periods around 1998–1999 (Drought 1) and 2002 (Drought 2) were highlighted within dark gray bars. Missing values of the weather station record at the test site were represented by light gray bars

All chronologies shared a strong growth reduction in 1999, corresponding to a low precipitation period from 1997 winter to 1999 (Figure 2d, first gray bar Drought 1, area colored red below normal expectation). The precipitation shortage was especially noticeable during the summers when most precipitation falls at the test site in a normal year. This drier‐than‐normal period was also remarkably warm (Figure 2b), causing a severe moisture deficit that resulted in reduced growth for all provenances (Figure 2a,c). The second pronounced growth reduction (Figure 2, Drought 2) occurred in 2002, coinciding with an unusually cold spring and a moderately dry summer during the year. Growth of provenances did not fully recover before this second drought period, and the response to the second drought might therefore be influenced by the first drought period as well. This second drought in 2002 was shorter than the first in 1999 at the study site, but it is still recorded as one of the most serious prairie droughts in the last several decades (Bonsal & Regier, 2007; Wheaton, Kulshreshtha, Wittrock, & Koshida, 2008). The average growth response of regional populations was reasonably precise and constant over time as indicated by standard errors of around 0.3 mm ring width.

### 3.2 Provenances and origin climate

Most geographic groups of provenances had distinct regional origin climates, except the Montane Cordillera region, which comprises a variety of climates due to complex topography (Figure 1b). The vectors represent the strength and directions of correlations of climate variables with the first two principal components. Provenances with high scores in the first principal component (PC1) (right side of Figure 1b) experienced a long growing season (MAT, MCMT, opposite DD < 0) and high moisture availability (MAP, MSP, PAS, opposite SHM). Provenances that score high for the second principal component (PC2, y‐axis of Figure 1b) experienced low growing season temperature variables (MWMT, DD > 5, FFP). Together, PC1 and PC2 explained 82.5% of total variance in climate variables.

The coldest and driest climate of the sample design was found in the Boreal Cordillera and Boreal Plains ecoregion of western Canada (Figure 1, Table 1). The eastern neighboring area, the Boreal Shield ecoregion comprising Ontario and Quebec sources, showed similar temperature conditions but twice the precipitation, which results in a lower annual moisture deficit. Maritime Mixedwood provenances from the east coast received the highest precipitation, the smallest moisture deficit, and the warmest winters. The southeastern Temperate Mixedwood ecoregion of southern Ontario had high annual moisture deficits due to high temperature. Montane Cordillera of the west coast was characterized by maritime climate with cool summers, relatively mild winters, and a low continentality score.

**Table 1:** Climate conditions for the 1961–1990 normal period for the provenance origins, summarized by regions as shown in Figure 1, with standard deviations shown in parentheses, as well as climate conditions at the Calling Lake test site in AB

Location	MAT (°C)	MWMT (°C)	MCMT (°C)	TD (°C)	MAP (mm)	MSP (mm)	CMD (mm)
Calling Lake (field site)	0.6	15.6	−18.0	33.6	491	336	150
Boreal Cordillera (*n* = 4)	−3.3 (1.3)	13.7 (1.2)	−22.6 (3.3)	36.3 (4.3)	310 (46)	193 (19.6)	201 (43.5)
Boreal Plains (*n* = 9)	−0.6 (1.6)	17.0 (1.4)	−21.7 (2.5)	38.7 (2.3)	450 (66)	291 (51.3)	177 (30.4)
Boreal Shield (*n* = 9)	0.7 (1.0)	16.5 (0.8)	−16.9 (2.5)	33.5 (2.9)	922 (179)	468 (66.8)	66 (52.6)
Maritime Mixedwood (*n* = 3)	5.0 (0.5)	17.4 (0.7)	−6.2 (0.8)	23.5 (1.3)	1,246 (146)	463 (31.1)	52 (16.0)
Montane Cordillera (*n* = 4)	1.2 (1.6)	13.4 (0.7)	−12.2 (3.5)	25.6 (3.5)	702 (250)	290 (77.1)	174 (66.0)
Temperate Mixedwood (*n* = 4)	5.5 (0.6)	20.1 (0.3)	−10.9 (1.6)	31.0 (1.6)	832 (11)	384 (19.2)	192 (10.7)

Note

The climate variables include mean annual temperature (MAT); mean warmest month temperature (MWMT); mean coldest month temperature (MCMT); temperature difference is a measure of continentality (TD); mean annual precipitation (MAP); mean summer precipitation (MSP); and climatic moisture deficit (CMD).

### 3.3 Regional population differentiation

Best linear unbiased estimates (BLUEs) of regional population means were estimated with generalized linear mixed models (GLMMs) for survival rate, and linear mixed models (LMMs) for all other traits (Table 2). Conforming to the expectations that local sources would be best adapted to the climate of the planting site, provenances from the Boreal Plains had the lowest mortality and showed good growth performance, with one of the highest height and diameter values measured after 32 and 27 growing seasons, respectively. Populations from Temperate Mixedwood (southern Ontario) and Boreal Shield (Ontario and Quebec) showed even better growth than local sources with the latter one originating from regions approximately 5°C warmer and double the annual precipitation compared to the test site (Table 1). The excellent growth performance of the nonlocal sources from the Boreal Shield did not significantly compromise survival (Table 2). In contrast, the sources close to the west coast and east coast had inferior growth performance and low survival rates at the central test site.

**Table 2:** Growth performance and drought response of white spruce provenances from six regions tested at a central provenance trial in Calling Lake, AB. Height and diameter at 1.3 m of six white spruce populations after 32 and 27 years of growth, respectively

Region	Growth traits			Drought tolerance traits				
	Height 32	DBH 27	Survival rate	Resistance 1999	Recovery 1999	Resistance 2002	Recovery 2002	Resilience
Boreal Cordillera	546 (55)^a^	63 (8)^a^	0.97 (0.02)^ab^	0.69 (0.04)^a^	1.70 (0.14)^a^	0.64 (0.06)^a^	1.73 (0.17)^ab^	1.10 (0.07)^a^
Montane Cordillera	700 (56)^b^	84 (8)^b^	0.84 (0.07)^ab^	0.60 (0.04)^ac^	1.77 (0.15)^ad^	0.53 (0.06)^a^	2.14 (0.12)^b^	0.95 (0.07)^a^
Maritime Mixedwood	767 (64)^b^	79 (9)^ab^	0.78 (0.10)^b^	0.60 (0.05)^ac^	1.85 (0.16)^abd^	0.69 (0.07)^a^	1.62 (0.13)^a^	1.07 (0.07)^a^
Boreal Plains	908 (37)^c^	116 (5)^c^	0.97 (0.01)^a^	0.50 (0.03)^bc^	2.17 (0.11)^bcd^	0.63 (0.05)^a^	1.72 (0.09)^a^	1.04 (0.05)^a^
Boreal Shield	957 (37)^c^	117 (5)^c^	0.95 (0.02)^ab^	0.44 (0.03)^b^	2.26 (0.11)^bc^	0.55 (0.05)^a^	1.78 (0.09)^a^	0.96 (0.05)^a^
Temperate Mixedwood	966 (55)^c^	122 (8)^c^	0.89 (0.05)^ab^	0.44 (0.04)^bc^	2.43 (0.14)^c^	0.67 (0.06)^a^	1.71 (0.12)^a^	1.04 (0.07)^a^

Note

Drought indicator values (larger values are better) for resistance, recovery of two drought years, and resilience for overall disturbances. Best linear unbiased estimates (BLUEs) and standard error of the means (in parentheses) were estimated with generalized linear mixed models (GLMMs) for survival rate, and linear mixed models (LMMs) for all other traits. Different superscript letters behind the values indicate significant differences between groups (*α* = 0.05).

### 3.4 Population response to drought

Regional population differentiation was also readily apparent in annual growth increments with the local provenances, Boreal Shield and Temperate Mixedwood sources being the best performers during the pre‐ and postdisturbance periods (Figure 2a). During the drought periods, provenances from all regions behaved similarly with reduced ring width increments, but the decreased measurements in percent of the predisturbance productivity (resistance) were inversely proportional to the normal growth rates before disturbance: 31% loss of growth (i.e., 69% resistance in 1999) for the most resistant Boreal Cordillera sources versus over 56% reduction for the sources from the Temperate Mixedwood and Boreal Shield regions (Table 2). For the 2002 drought, the percent reduction in growth was less differentiated (Figure 2), with no significant regional differences in resistance (Table 2). Provenances from all regions recovered approximately to their predisturbance levels, indicating no permanent damage after two consecutive drought events. Unlike the 1999 event, the 2002 growth reduction showed no statistically significant regional differences of resistance or recovery.

### 3.5 Genetic adaptation to local climate conditions

**Figure 3:**
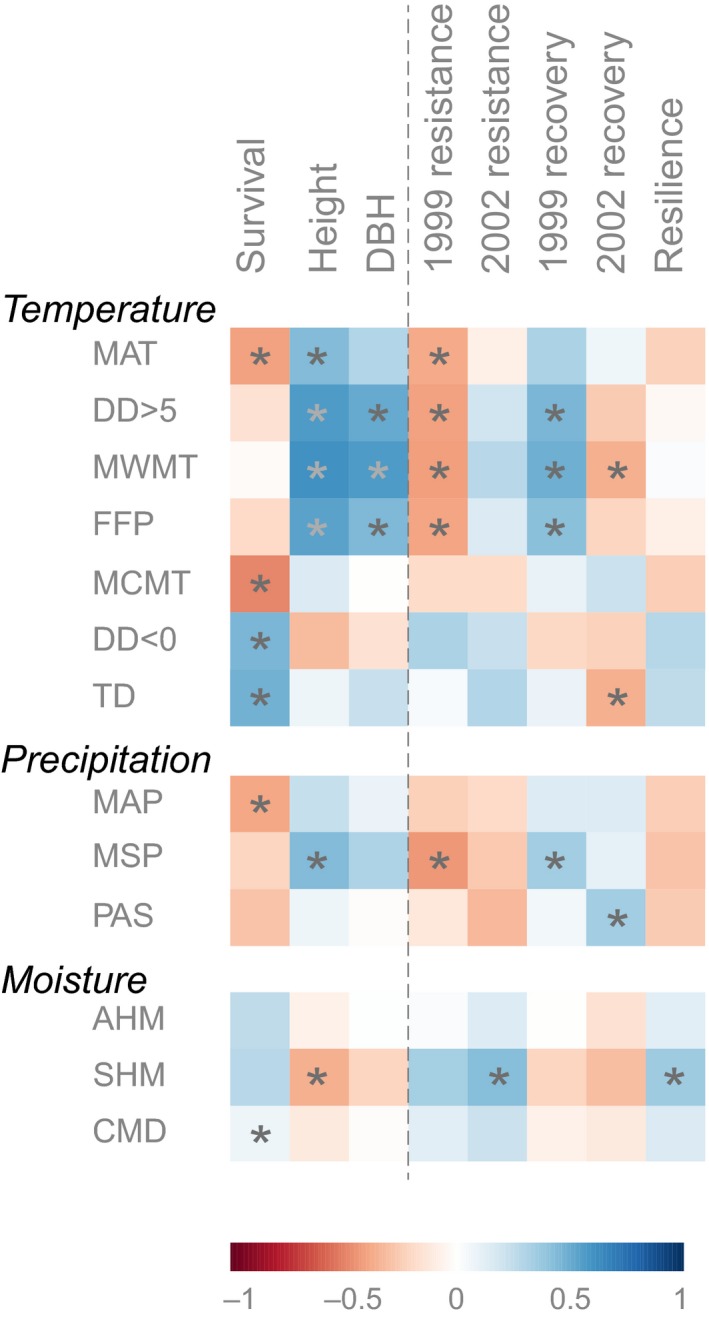
Correlations among the climate of provenance origin and growth measurements at the field site and drought metrics calculated from tree ring analysis. Significant correlations after adjustment for multiple inference are highlighted with asterisks (*p* < .05). Climate variables include mean annual temperature (MAT), chilling and growing degree‐days (DD < 0, DD > 5), mean temperature for the warmest (MWMT) and coldest month (MCMT), frost‐free period (FFP), continentality (TD), growing season precipitation (May to September) (MSP), annual precipitation (MAP), precipitation as snow (PAS), annual and summer heat–moisture index (AHM, SHM), and Hargreaves climatic moisture deficit (CMD). DBH abbreviates diameter at breast height

Seed sources originating from warm and wet origin climate showed superior growth potential at our test site (Figure 3). Height and diameter positively correlated to long and moist growing seasons of the provenance origin climate. In contrast, a high survival rate was closely associated with dry environments and cold, prolonged winters. Survival rate showed no correlations with climate variables strongly associating with height and diameter. Resistance for the 1999 drought was negatively related to growing season length and summer precipitation of the provenance origin climate, which mirrored associations with height and diameter. Therefore, trees from warm and moist climate conditions grow well, but also experience the proportionally largest growth reductions under drought conditions. For the climate conditions during the 2002 drought period, characterized by a cool spring and dry summer, correlations among provenance origin climate and drought metrics were weak (*r* < 0.5) (Figure 3). Provenances from continental climates (dry, hot summers with cold winters) maintained better growth under the 2002 climate conditions. Since the recovery index is an inverse calculation of the resistance values under full recovery, matching, but opposite correlations for recovery and resistance indices were observed for each disturbance. No genetic differentiation and no strong associations were identified between resilience and the origin climate of provenances, indicating that provenances have comparable drought resilience, with all regional populations avoiding permanent damage from the drought episodes.

## 4 DISCUSSION

The general expectation for widespread forest trees that occur over a wide range of environmental conditions is to find genetic population differentiation in growth and adaptive traits. Local sources are expected to have the highest fitness and often also the highest growth rates (Morgenstern, 1996). Our data for white spruce partially conform to these expectations. Local sources from the Boreal Plains ecoregion were among the top performers. Boreal Shield and Temperate Mixedwood sources from eastern Canada slightly outperformed the local sources, but for the Temperate Mixedwood provenances, fitness appears to be compromised based on the lower survival rates. Growth measurements generally conform to comparable trials in eastern Canada that also showed populations from the southern fringe of the boreal forest have the highest growth potential, even when transferred to northern test sites (Lu et al., 2014).

A possible explanation for this observation is that southern populations already lag behind their optimal niche due to climate warming that has occurred over the last several decades. Alternatively, adaptation to biotic factors may explain the superior growth potential of Temperate Mixedwood sources from southern Ontario. In warmer mixed forest ecosystems, competition for light from other species causes higher selection pressure for fast growth, causing individuals to allocate less resource to survival adaptations (Loehle, 1998). When such sources are moved northward beyond their optimal climatic niche, their fitness could be compromised as indicated by lower survival rates (Table 2). Similar to Andalo, Beaulieu, and Bousquet (2005), we find that population differentiation in growth and survival is related to the temperature of the provenance origin. Here, the winter temperature of the origin was correlated with survival and the growing season length was associated with growth (Figure 3).

The most notable result from this range‐wide provenance experiment is that no obvious adaptation in growth and survival was observed in response to various precipitation regimes (Figure 3), conforming to the finding of equal drought resilience even when provenances from throughout the natural range were exposed to extreme drought conditions that severely impacted the annual growth of all provenances (up to over 50% growth reduction compared to a normal year). While resistance and recovery metrics significantly varied among populations (Table 2), no crossover interactions were observed where a provenance with lower growth under favorable moisture conditions exceeds productive provenances under drought conditions (Figure 2). No population was able to maintain a substantially higher level of productivity under drought constraints. This implies that the species as a whole may lack genetic variation and adaptive capacity to maintain growth under drought. That said, our sampling design did not cover a sufficient amount of samples from isolated or marginal populations at the southern boreal fringe, where such adaptation may still be found.

Nevertheless, our results are different to what has been observed for other boreal and temperate conifers. For example, lodgepole pine, which overlaps with white spruce in the west, lacks adaptive capacity for drought conditions in northern populations of the Boreal Cordillera but shows distinct adaptations to drought in southern populations (Montwé et al., 2016). Douglas fir showed a trade‐off between growth performance and drought tolerance, with the provenances from the warmest and driest origins showing the highest drought tolerance (Bansal, Harrington, Gould, & St Clair, 2015; Eilmann et al., 2013; Montwé et al., 2015). Norway spruce also shows strong adaptive genetic variance in drought responses, with populations from the central and southeastern portion of the range exhibiting high resistance (Trujillo‐Moya et al., 2018).

These results have implications for seed movement as an adaptation strategy for climate change. Human‐assisted migration as part of regular reforestation programs has been proposed to match planting stock with anticipated new environments, as populations become progressively mismatched with the climate to which they are adapted (Aitken & Bemmels, 2016; Bertrand et al., 2011; Lenoir et al., 2008; Pedlar et al., 2011; Peters & Darling, 1985). As an adaptation strategy, assisted migration prescriptions are now being implemented to address maladaptation of forests in the boreal North (Ste‐Marie, 2011, 2014). The provinces of British Columbia, Alberta, Ontario, and Quebec updated seed transfer rules to facilitate assisted migration through seed movement in their regular reforestation programs (O’Neill et al., 2017). However, genetic differentiation among populations is a necessary requirement for assisted migration prescriptions to be effective. If no genetic differentiations can be identified in drought resilience or for maintenance of growth under drought conditions, seed movement may have not been able to improve drought tolerance in areas that are expected to have high water deficits.

**Figure 4:**
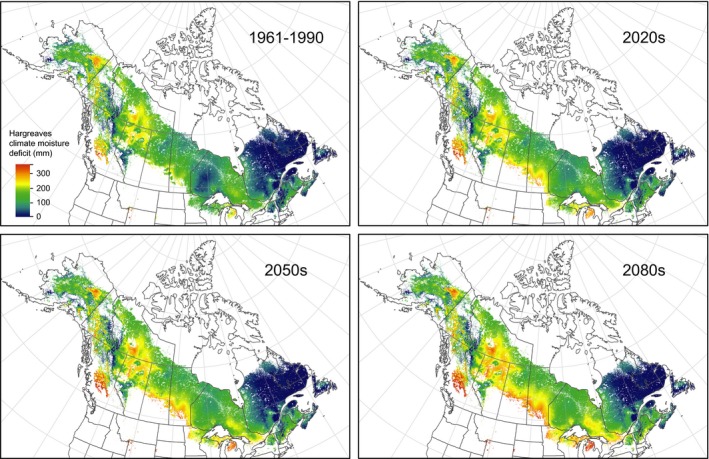
Hargreaves climatic moisture deficit (CMD) for the current distribution of white spruce. The 1961–1990 normal represents a historic reference period, and the 2020s, 2050s, and 2080s are Ensemble multi‐model (CMIP5) projections according to Wang et al. (2016)

To provide additional context for interpreting the results of this common garden experiment, we provide maps of moisture deficits for the species range of white spruce for the 1961–1990 normal period and projected future conditions (Figure 4), generated with the same methodology as for obtaining moisture deficit estimates for the test site and provenance locations (Table 1). Generally, climate moisture deficits for the range of white spruce ranges from values close to zero in the northeast to values of around 250 mm in portions of its western range. Ensemble projections from CMIP5 global circulation models according to Wang et al. (2016) show that 250 mm moisture deficits are exceeded along the southern fringe of the species distribution as well as for large parts of Alberta and the Northwest Territories (Figure 4, brown hues). This is substantially drier than at the test site during the growing period (152 mm). Given the observed growth response during drought years, it seems likely that white spruce growth throughout the generally drier western portion of the boreal forest may on average be reduced by a substantial amount (up to 50% observed in this experiment), without the option to adapt to climate change by selection of drought‐tolerant populations in reforestation programs.

While we did not observe drought‐induced mortality or dieback at this specific experiment, water deficits limit the range of white spruce at the southern fringe (Chhin & Wang, 2008; Chhin et al., 2004) and have caused mortality of white spruce in the west (Peng et al., 2011). In comparison with five other boreal species, D'Orangeville et al. (2018) found that white spruce appears to be especially prone to growth reductions under all climate simulations above 2°C warming. While white spruce did not show massive mortality in response to drought as other species have (Worrall et al., 2013), their general fitness appears compromised. Searle and Chen (2017) showed a long‐term trend towards reduced abundance of late‐successional conifers (represented by 93% white spruce), with the strongest reductions observed in western Alberta, and they attributed the trend to warming and increases in CO2.

For the eastern half of the species range, projections up to the 2080s do not appear to exceed values of 250 mm in moisture deficits. Results from the provenance trial further show that eastern provenances generally grow well under these conditions. In the northeast, predicted climate moisture deficits are generally above 100 mm, substantially wetter than the planting site (152 mm). These results support the view that northeastern regions of Canada appear to be a likely refugium for boreal forest tree species in a warming climate (D'Orangeville et al., 2016), while the western part of the boreal forest is prone to growth reductions under warming environment because of moisture sensitivity. For resource managers and policy makers, our results suggest that planning for widespread declines of western boreal white spruce forests may be unavoidable in many parts of the species range. Specifically, moving seed sources northward in the western boreal region to counter anticipated moisture deficits may not be an effective climate change adaptation strategy.

## 5 CONCLUSIONS

The results highlight that population differentiation in adaptive capacity to climate environments can be species‐ and trait‐specific, and we provide a counterexample where assisted migration prescriptions may be ineffective to mitigate climate change impacts in the western boreal portion of the species range. The study also provides direct evidence that northeastern populations of white spruce may be safe from negative climate change impacts for the medium‐term future, with no permanent damage observed on northeastern populations when subjected to drought conditions at the test site that they would be unlikely to experience in their native region even under pessimistic climate change projections by the 2080s. In contrast, the fate of western populations is more difficult to assess based on direct evidence from this study. We observed strong growth reductions, but no permanent damage arising from droughts in a region where the average annual water deficit is around 150 mm. However, this value is predicted to be exceeded by the 2050s throughout the western half of the species distribution. Planning for widespread declines of western boreal white spruce forests may be unavoidable where water deficits limit the species productivity. This may lead to large‐scale mortality as recently observed for lodgepole pine in western boreal forests. Such subcontinental‐scale ecosystem disruptions are likely too large to be effectively mitigated through forest management or reforestation prescriptions.

## CONFLICT OF INTEREST

None declared.

## Data Availability

Data for this study are available at the Dryad Digital Repository: https://doi.org/10.5061/dryad.n42p4k7. (Sang, Sebastian‐Azcona, Hamann, Menzel, & Hack, 2019)
